# Toward immortality: Natural variation in maize *PROTEIN L-ISOASPARTYL O-METHYLTRANSFERASE 1* regulatory region shapes seed vigor and longevity

**DOI:** 10.1093/plcell/koaf227

**Published:** 2025-09-23

**Authors:** Nitin Uttam Kamble

**Affiliations:** Assistant Features Editor, The Plant Cell, American Society of Plant Biologists; Indian Institute of Science Education and Research, Thiruvananthapuram 695551, India

The seed, in its seeming simplicity, is a vessel of potential life. It carries within its quiescent structure the encoded memory of countless generations of evolutionary refinement, awaiting only the right conditions to awaken growth. Orthodox seeds (Roberts 1973) have evolved the remarkable ability to tolerate desiccation, allowing them to survive under dry conditions for years and even, in some cases, for millennia. Nevertheless, deterioration and aging are inevitable processes that gradually erode seed germination potential.

Studies indicate that the regulation of seed germination potential (longevity, vigor, and viability) is a highly complex trait requiring the coordinated functioning of multiple proteins. However, proteins are inherently vulnerable to structural and functional damage, which is exacerbated under stress, prolonged storage, or aging. Research has demonstrated the critical role of the protein repair enzyme PROTEIN L-ISOASPARTYL O-METHYLTRANSFERASE (PIMT) in regulating seed germination potential of seeds (Ogé et al. 2008; Kamble and Majee 2022; Zhang et al. 2023). Despite these advances, many preferred substrate proteins of PIMT remain unidentified, and the functional consequences of natural variation in *PIMT* regulatory regions are still largely unexplored.

In an exciting new work, Zhang et al. (2025) shed light on the functional significance of natural variation in the regulatory regions of *PIMT* for seed vigor in maize. The maize lines Zheng58 and Chang7-2 displayed contrasting seed vigor profiles. Chang7-2 exhibited greater and more robust seed germination than Zheng58 (indicative of better seed vigor), both with and without accelerated aging (AA; 95% relative humidity at 45 °C for 4 days) treatment.

Given the well-established role of PIMT in seed vigor, which in maize (and in plants generally) is encoded by 2 genes, *ZmPIMT1* and *ZmPIMT2*, the authors analyzed the mRNA and protein levels for both the genes in these lines under normal and AA conditions. The results revealed differential expression of *ZmPIMT1* between the two lines, while *ZmPIMT2* levels remained consistent. Notably, Chang7-2 accumulated greater amounts of *ZmPIMT1* transcript and protein than Zheng58.

Using a Zhengdan 958 recombinant inbred line population derived from a cross between Zheng58 and Chang7-2, the authors performed expression genome-wide association analysis and identified a major SNP within the regulatory region of *ZmPIMT1*. Amplification of this region revealed polymorphisms, with most recombinant inbred lines carrying amplicons like Chang 7-2, while others displayed amplicons resembling Zheng 58. Sequencing this region identified a 2316-bp insertion 222 bp upstream of the start codon in *ZmPIMT1* (Figure). Functional validation using *zmpimt1* knockdown and *ZmPIMT1* overexpression lines confirmed that elevated expression of *PIMT1* enhances seed vigor.

**Figure.**
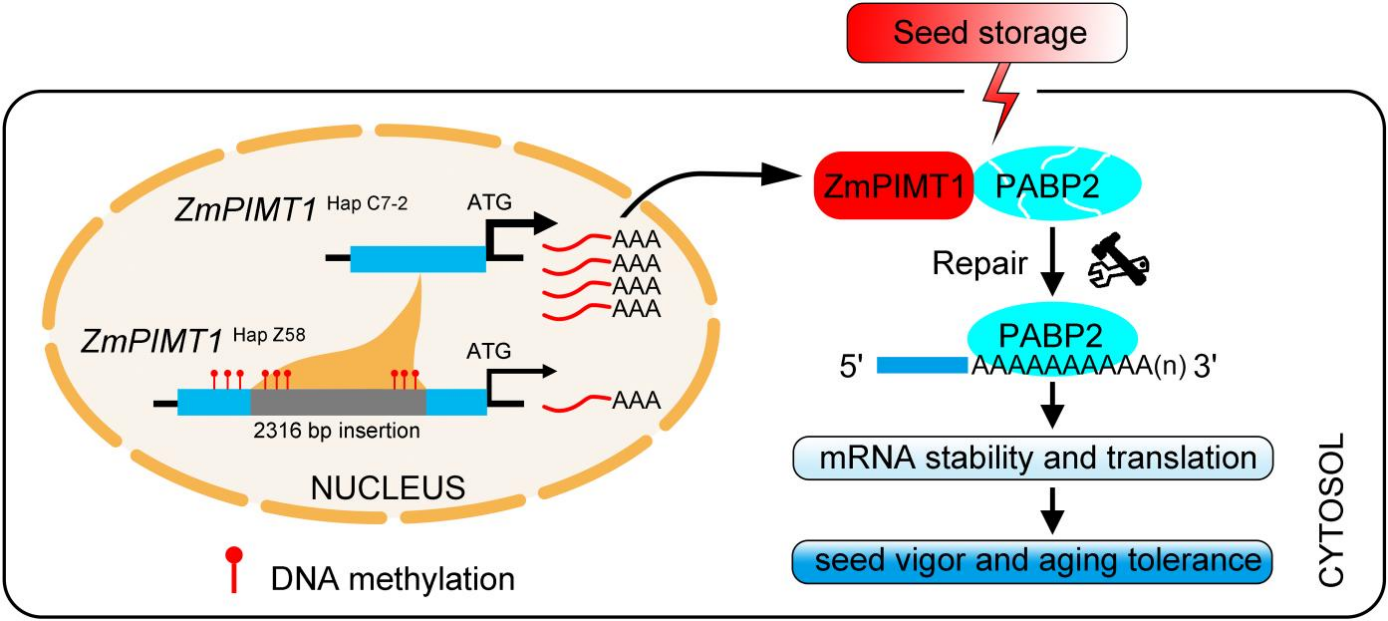
Regulation of seed vigor by PROTEIN L-ISOASPARTYL O-METHYLTRANSFERASE (PIMT) in maize. *ZmPIMT1* promoter shows natural variation (*ZmPIMT1^HapC7–2^*) having a 2316-bp fragment deleted, increasing *ZmPIMT1* expression by avoiding DNA methylation of the promoter. Higher PIMT1 maintains the mRNA binding ability of ZmPABP2 by repairing the isoAsp modifications to positively regulates seed vigor. Reprinted from Zhang et al. (2025), Figure 9.

To explore the molecular mechanisms underlying *ZmPIMT1*-mediated seed vigor, the authors performed co-immunoprecipitation experiments and identified several interacting putative substrate proteins. Among these, multiple Class II polyadenylate binding proteins (PABPs) emerged as strong candidates. Localization studies showed that ZmPABP2 and ZmPIMT1 co-localize to the cytosol and physically interact, as confirmed by in vivo co-immunoprecipitation. Importantly, Arabidopsis mutants lacking Class II PABPs also exhibited reduced seed vigor when naturally aged or following AA treatment. Using temperature-related intensity change assays, the authors demonstrated that PABPs retain their poly(A)-mRNA binding capability in the presence of PIMT1 (Figure). Since PABPs are essential for RNA stabilization, the authors further conducted RNA-seq and ribosome nascent-chain complex sequencing in *zmpimt1* embryos with and without AA treatment and observed significant alterations in mRNA abundance and translation efficiency for numerous genes compared with controls.

In summary, this study elucidates the natural variation in the regulatory regions of *ZmPIMT1* and reveals the molecular mechanisms underpinning seed vigor regulation in maize via the PIMT1-PABP module (Figure). Understanding these regulatory networks and the ability to manipulate them through breeding and biotechnology holds significant potential for developing seeds with enhanced agronomic traits, improving agricultural productivity, securing food systems, and optimizing nutritional content to meet the demands of a growing population and expanding industrial applications.

## Recent related articles in *The Plant Cell*:

- Tang et al. (2025) described the antagonizing role of Arabidopsis evening complex proteins toward ABI3 and ABI5 to temporally regulate abscisic acid signaling and seed germination.
- Du et al. (2024) described the role of Arabidopsis U-box E3 ubiquitin ligase PUB35 in regulation of ABA signaling through AFP1-mediated degradation of ABI5 during seed germination.
- Mei et al. (2023) reported that auxin contributes to jasmonate-mediated regulation of abscisic acid signaling during Arabidopsis seed germination.
- Varshney et al. (2023) demonstrated the regulation of Arabidopsis JAZ proteins during seed maturation and seed vigor, independently of jasmonic acid-isoleucine, by the F-box protein SKP1-INTERACTING PARTNER 31.

## Dive Curated Terms

The following phenotypic, genotypic, and functional terms are of significance to the work described in this paper:

PIMT1 Gramene: AT3G48330
PIMT1 Araport: AT3G48330

ABI5 Gramene: AT2G36270
ABI5 Araport: AT2G36270

ABI3 Gramene: AT3G24650
ABI3 Araport: AT3G24650

PUB35 Gramene: AT4G25160
PUB35 Araport: AT4G25160
